# Comparison of refugee patients with cystic fibrosis and their counterpart children from Turkey during the war

**DOI:** 10.1007/s00431-024-05431-8

**Published:** 2024-01-24

**Authors:** Aslı İmran Yılmaz, Sevgi Pekcan, Tuğba Şişmanlar Eyüboğlu, Melih Hangül, Hüseyin Arslan, Ayşe Ayzıt Kılınç, Haluk Çokuğraş, Elif Arık, Özlem Keskin, Ali Özdemir, Murat Ersoy, Ali Ersoy, Mehmet Köse, Beste Özsezen, Gökçen Ünal, Ömür Ercan, Saniye Girit, Sinem Can Oksay, Yasemin Gökdemir, Bülent Karadağ, Velat Şen, Erkan Çakır, Hasan Yüksel, Merve Nur Tekin, Ayşe Tana Aslan

**Affiliations:** 1 Department of Pediatric Pulmonology, Faculty of Medicine, Necmettin Erbakan University, Konya, Turkey; 2 Department of Pediatric Pulmonology, Faculty of Medicine, Gazi University, Ankara, Turkey; 3 Pediatric Pulmonology, Adana City Training and Research Hospital, Adana, Turkey; 4 Department of Pediatric Pulmonology, Cerrahpaşa Faculty of Medicine, Istanbul University - Cerrahpasa, Istanbul, Turkey; 5 Department of Pediatric Allergy and Immunology, Faculty of Medicine, Gaziantep University, Gaziantep, Turkey; 6 Department of Pediatric Pulmonology, Mersin City Training and Research Hospital, Mersin, Turkey; 7 Department of Pediatric Pulmonology, Faculty of Medicine, Erciyes University, Kayseri, Turkey; 8 Department of Pediatric Pulmonology, Şanlıurfa Training and Research Hospital, Şanlıurfa, Turkey; 9 Department of Pediatric Pulmonology, Faculty of Medicine, Istanbul Medeniyet University, Istanbul, Turkey; 10 Department of Pediatric Pulmonology, Marmara University Pendik Training and Research Hospital, Istanbul, Turkey; 11 Department of Pediatric Pulmonology, Faculty of Medicine, Dicle University, Diyarbakır, Turkey; 12 Department of Pediatric Pulmonology, Liv Hospital, Istinye University, Istanbul, Turkey; 13 Department of Pediatric Pulmonology, Faculty of Medicine, Celal Bayar University, Manisa, Turkey; 14 Department of Pediatric Pulmonology, Faculty of Medicine, Ankara University, Ankara, Turkey

**Keywords:** Children, Cystic fibrosis, Refugee, War

## Abstract

Since the outbreak of the Syrian civil war in 2011, the population of Arab refugees in Turkey has rapidly increased. While cystic fibrosis (CF) is believed to be rare among Arabs, recent studies suggest it is underdiagnosed. This study aims to present the demographic, clinical, and genetic characteristics of CF patients among Arab refugees in Turkey. Additionally, a comparison is made between the findings in the National CF Registry 2021 in Turkey (NCFRT) and the refugee CF patient group. The study included refugee patients between the ages of 0 and 18 years who were diagnosed with CF and received ongoing care at pediatric pulmonology centers from March 2011 to March 2021. The study examined demographic information, age at diagnosis, age of diagnosis of patients through CF newborn screening (NBS), presenting symptoms, CF transmembrane conductance regulator (CFTR) mutation test results, sputum culture results, weight, height, and body mass index (BMI) z score. Their results were compared with the NCFRT results. The study included 14 pediatric pulmonology centers and 87 patients, consisting of 46 (52.9%) boys and 41 (47.1%) girls. All of the patients were Arab refugees, with 80 (92%) being Syrian. All the patients were diagnosed in Turkey. The median age at diagnosis of patients was 22.33 (interquartile range, 1–258) months. The median age of diagnosis of patients through NBS was 4.2 (interquartile range, 1–12) months. The median age of older patients, who were unable to be included in the NBS program, was 32.3 (interquartile range, 3–258) months. Parental consanguinity was observed in 52 (59.7%) patients. The mutation that was most frequently found was F508del, which accounted for 22.2% of the cases. It was present in 20 patients, constituting 32 out of the total 144 alleles. There was a large number of genetic variations. CFTR genotyping could not be conducted for 12 patients. These patients had high sweat tests, and their genetic mutations could not be determined due to a lack of data. Compared to NCFRT, refugee patients were diagnosed later, and long-term follow-up of refugee CF patients had significantly worse nutritional status and pseudomonas colonization.

*  Conclusion*: Although refugee CF patients have equal access to NBS programs and CF medications as well as Turkish patients, the median age at diagnosis of patients, the median age of diagnosis of patients through NBS, their nutritional status, and Pseudomonas colonization were significantly worse than Turkish patients, which may be related to the difficulties of living in another country and poor living conditions. The high genetic heterogeneity and rare mutations detected in the refugee patient group compared to Turkish patients. Well-programmed NBS programs, thorough genetic studies, and the enhancement of living conditions for refugee patients in the countries they relocate to can have several advantages such as early detection and improved prognosis.

**Table:** 

**What is Known:** *• Children who have chronic diseases are the group that is most affected by wars.* *• The outcome gets better with early diagnosis and treatment in patients with Cystic Fibrosis (CF).*	
**What is New:** *• Through the implementation of a newborn screening program, which has never been done in Syria previously, refugee patients, the majority of whom are Syrians were diagnosed with cystic fibrosis within a duration of 4 months.* *• Despite equal access to the newborn screening program and CF medications for both Turkish patients and refugee patients, the challenges of living in a foreign country have an impact on refugees.*

**What is Known:**

*• Children who have chronic diseases are the group that is most affected by wars.*

*• The outcome gets better with early diagnosis and treatment in patients with Cystic Fibrosis (CF).*

**What is New:**

*• Through the implementation of a newborn screening program, which has never been done in Syria previously, refugee patients, the majority of whom are Syrians were diagnosed with cystic fibrosis within a duration of 4 months.*

*• Despite equal access to the newborn screening program and CF medications for both Turkish patients and refugee patients, the challenges of living in a foreign country have an impact on refugees.*

## Introduction

Cystic fibrosis (CF) is an autosomal recessive chronic disease caused by mutations in the CF transmembrane conductance regulator (CFTR) gene. The CFTR protein affects the ion channel function on the apical surface of epithelial cells, causing a change in mucus rheology and producing mucus with a thick sticky consistency that is difficult to clear. This leads to obstruction in the channels, and high susceptibility to infections develops. CF is distributed worldwide and is most common (1 in 2500 Caucasians) in Northern Europeans [1]. CF has been included in the newborn screening (NBS) program in Turkey since January 1, 2015. It is thought to be rare in the Arab population, but its exact prevalence is not known. In Arabs, CF is not properly diagnosed because the symptoms resemble those of malnutrition, which is common in societies with low socioeconomic status during times of war. Furthermore, the occurrence of CF may be higher in this population due to the high rate of consanguinity.

One of the most important problems today is regional wars. The number of refugees, who constitute a minority in Turkey, has witnessed a rapid increase following the onset of the civil war in neighboring Syria in 2011. Millions of adults, children, and older individuals have migrated, particularly to neighboring countries, such as Turkey, Lebanon, Jordan, Iraq, and Egypt. It has now been 13 years since the political crisis began in Syria. As stated in the 2023 report by the United Nations Refugee Agency (UNHCR), 64.5% of Syrian refugees are currently residing in Turkey. Unfortunately, 51% of these refugees are minors aged under 18 years [2]. Challenges related to vaccination, protection against infectious diseases, and the psychological impact of war make children the most vulnerable group in times of conflict. Children with chronic diseases need to be able to access and administer their medicines on time because this has an impact on their life expectancy. The Turkish Government has been addressing the fundamental requirements of refugees, which include housing, healthcare, and food. All newborn refugees have benefited from the NBS program that was implemented in Turkey. These patients can be examined free of charge in health institutions and have access to medical treatment. The Turkish Disaster and Emergency Management Presidency offers assistance for regular education and basic daily necessities. Although there is a minority group residing in tent cities, the majority of refugees prefer to settle in areas near the Syrian border and in major cities [3, 4].

This study aimed to present the demographic, clinical, and genetic characteristics of refugee patients with CF in Turkey and to compare them with those of children with CF who have been registered in the National CF Registry in Turkey 2021 (NCFRT) report [5, 6].

## Materials and methods

### Study design

This was a retrospective, multicenter study. Approval was obtained from the Necmettin Erbakan University Faculty of Medicine Ethics Committee (2021/3502).

### Setting

All pediatric CF centers were invited to participate in the study, and a total of 14 centers that provide care for CF patients who are refugees were included in the study. These centers include the following: Necmettin Erbakan University Faculty of Medicine, Gazi University Faculty of Medicine, Adana City Training and Research Hospital, Istanbul University-Cerrahpasa, Cerrahpaşa Faculty of Medicine, Mersin City Training and Research Hospital, Erciyes University Faculty of Medicine, Şanlıurfa Training and Research Hospital, Istanbul Medeniyet University Faculty of Medicine, Marmara University Pendik Training and Research Hospital, Dicle University Faculty of Medicine, Istinye University, Liv Hospital, Celal Bayar University Faculty of Medicine, Ankara University Faculty of Medicine, Department of Pediatric Pulmonology, and Gaziantep University Faculty of Medicine, Department of Pediatric Allergy and Immunology.

### Population

The study included refugee children aged 0–18 years with CF who were monitored in pediatric chest disease clinics in Turkey from March 2011 to March 2021. According to the Geneva Refugee Convention, a refugee is defined as an individual who has a well-founded fear of persecution based on their race, religion, nationality, membership in a particular social group, or political opinion. This fear leads them to leave their country and either not return or not want to return due to this fear [1, 7]. The status of being a refugee is recognized under the law, and Turkey became a signatory to this convention in 1961. However, immigrants and asylum-seekers were not considered in the study.

Patients who were diagnosed as having CF according to the consensus report created by the American Cystic Fibrosis Foundation were included in the study. It has been stated that if one or more typical CF findings are present, along with a history of a sibling with CF or a positive NBS, CF can be diagnosed by demonstrating CFTR anomalies under laboratory conditions. This can be done by observing an increased chloride concentration in sweat tests, two mutations in the CFTR gene, or abnormal nasal epithelial ion transport [8, 9].

### Data collection

Patient information was electronically scanned from patient files. The study examined demographic information, age at diagnosis, age at diagnosis after NBS implementation in Turkey, symptoms at presentation, results of CFTR mutation tests, multiplex ligation-induced probe amplification (MLPA) analysis, results of sputum cultures, weight, height, and body mass index (BMI) z scores (for ages 2–18 years) calculated using the World Health Organization software (specifically the January 2011 anthropometric calculator).

The protocol used for CF screening in NBS in Turkey is to check for immunoreactive trypsinogen (IRT/IRT) in a dry blood spot twice, with a 1-week gap between tests. It is considered positive if the first test showed IRT > 90mg/L or the second test showed IRT > 70mg/L. A positive screening test must be confirmed by either a positive sweat test (sweat chloride > 60mmol/L) or the presence of two mutations known to cause CF [10].

Vitamin D deficiency was defined as vitamin D level below 20 mg/dl.

### Statistical analysis

Analyses were performed using the SPSS 25.0 package program. The variables were investigated using visual (histogram, probability plots) and analytic methods. The Kolmogorov–Smirnov/Shapiro–Wilk test was used to determine whether data were normally distributed. Descriptive analyses are presented using proportions, means, standard deviation (SD), medians, and interquartile range (IQR) as appropriate. A one-sample *t*-test was used to analyze the difference between the measurement values of the two groups. Chi-square test analysis was used for proportional comparisons. *P*-values less than 0.05 were considered statistically significant.

## Results

A total of 87 patients from 14 CF centers participated in the study. Out of these, 46 (52.9%) were boys and 41 (47.1%) were girls. All of the patients were Arab refugees, and 80 (92%) were Syrian nationals. All the patients were diagnosed in Turkey. The median age at diagnosis was 22.33 (IQR, 0.7–258) months. Thirty-one (35.6%) refugee patients were born after 2015, and all were diagnosed through the NBS. The median age of diagnosis of patients through NBS was 4.2 (IQR, 1–12) months. The median age of older patients, who were unable to be included in the NBS program, was 32.3 years (IQR, 3–258). There was a significant difference in the age at diagnosis between individuals diagnosed with NBS and others (*p* < 0.05). When looking at the admission symptoms, 32 (34.4%) had recurrent lower respiratory tract infections, (*n* = 8, 9.1% each) had growth retardation, pseudo-Bartter’s syndrome, and meconium ileus. Parental consanguinity was present in 52 (59.7%) patients. Additionally, 23 (26.4%) had a sibling with a history of CF. Pancreatic insufficiency was present in 67 (77%) of patients. The mean initial IRT value of patients who came from the NBS was 155.2 ± 52.5 (87, 280) ng/mL, and the second mean IRT value detected was 132 ± 51.5 (70, 273) ng/mL. The mean sweat test in patients with chloride demonstrated results of 85 ± 25.1 (60, 147) mmol/L. The median follow-up period was 46.6 (IQR, 2.5–82) months. Table 1 provides further demographic and clinical information.

**Table 1:** Demographic and clinical information of the refugee patients

	Refugee patients (*n* = 87)
Males *n* (%)	46 (52.9)
Famales *n* (%)	41 (47.1)
Median (IQR) age at diagnosis (months)	22.33 (0.7–258)
Median (IQR) current age (months)	66.7 (4–252)
Median (IQR) follow-up period (months)	46.6 (2.5–82)
Number of patients diagnosed through NBS *n* (%)	31 (35.6)
Median (IQR) age at diagnosis of patients through NBS (months)	4.2 (1–12)
Parental consanguinity *n* (%)	52 (59.7)
Pancreatic insufficiency *n* (%)	77 (88.5)
The initial IRT value ng/mL*	155.21 ± 52.58
Second IRT value ng/mL**	132.09 ± 51.51
The mean sweat test mmol/L***	85.09 ± 25.18
*Staphylococcus aureus colonization* (MSSA + MRSA) *n* (%)	25 (29.2)
*Pseudomonas aeruginosa colonization n* (%)	32 (36.8)
rhDNase *n* (%)	58 (66.7)
Pancreatic enzyme *n* (%)	76 (87.5)
Multivitamin *n* (%)	77 (88.5)

*IRT* immunoreactive trypsinogen, *rhDNase* recombinant human deoxyribonuclease, NBS newborn screening, *MSSA* methycilline sensitive *Staphylococcus aureus*, *MRSA* methycilline resistance *Staphylococcus aureus*

Normal value: * > 90 mg/L; ** > 70 mg/L; *** > 60 mmol/L

The mean vitamin D level was measured at 24.5 ± 3.1 (3–51.2) nmol/L in the patients. The mean height, weight, and BMI z score of the patients at the time of admission and their last visit are presented in Table 2.

**Table 2:** The height, weight, and *body mass index* (BMI) score follow-ups of the refugee patients at the time of admission and at their last visit

Z score	First admission	Last control
Mean weight ± sds	− 1.65 ± 1.85	− 1.8 ± 1.6
Mean height ± sds	− 0.13 ± 4.2	− 1.7 ± 1.5
Body mass index ± sds	− 1.26 ± 1.95	− 0.4 ± 4.4

*sds* standard deviation score

A total of 41 different variants were found. Homozygous mutations were found in 59 (78.6%) patients. The most commonly found variant, F508del, accounted for 22.2% of the total alleles (32 out of 144). There was a total of 20 (26.6%) patients identified as heterozygous and homozygous for this mutation. The second most common mutation was CFTRdel2-3, which accounted for seven cases (9.7%) (four of whom were siblings). We also discovered that almost all patients who carried this deletion in both copies of the gene experienced severe respiratory problems, Pseudomonas infection, pancreatic insufficiency, liver cirrhosis, a lower BMI, and more frequent failure to thrive. Additionally, they had high sweat test results and were diagnosed as having CF before the age of 1 year. Other variants that were detected included 2043delG, R352Q, 1677delTA, N1303K, L997F, I175V, S1118F, S1235R, 2184delA, 3121-1G > A, CFTRdel12-18, R352Q, Y1424Y, 2043delG, CFTRdel12-18, and I506V. Y1424Y and c.1911del variants were not found in NCFRT but were only detected in the refugee patient group. CFTR genotyping could not be conducted for 12 patients. These patients had high sweat tests, and their genetic mutations could not be determined due to a lack of data. The most frequently occurring genetic variants are presented in Table 3.

**Table 3:** The most common frequency occurrence of genetic variants in refugee patients

	Heterozygous gene variant *n* (%)	Homozygous gene variant *n* (%)	Total *n* (%)
F508del	8 (10.6)	12 (16)	20 (22.2)
Exon 2–3 deletion	0	7 (9.3)	7 (9.7)
1898 + 3A->G	2 (2.6)	3 (4)	5 (5.5)
W1282X	2 (2.6)	2 (2.6)	4 (4.1)
2183AA->G	0	3 (4.1)	3 (4.1)
c.1911del	0	3(4)	3 (4.1)

There was a significant difference in the median age at diagnosis between the NCFRT and refugee CF patient groups, and this time was significantly longer in refugee patients (22.3 vs. 4 months). When both groups were evaluated based on factors such as nutritional status and pseudomonas colonization, the refugee patient group had significantly poorer outcomes. Additionally, while of the frequency F508del mutation was found to be approximately the same in both groups during genetic evaluation, the second most common mutations differed. A comparison of demographic characteristics, the most common two mutations in CFTR, and the treatments used between refugees and NCFRT are given in Table 4.

**Table 4:** Comparison of refugee patients and NCFRT

	Refugee patients *n* = 87	NCFRT *n* = 1948	*P* value
Median (IQR) age at diagnosis (months)***	22.33 (1–258)	3 (0.9–492)	0.01*
Median (IQR) current age (months)***	66.7 (4–252)	100 (0.9–552)	0.01*
Median (IQR) age of patients diagnosed through NBS (months)***	4.2 (0.7–18)	1 (0.9–3)	0.01*
Median (IQR) weight (z score)***	− 1.8 (− 5.7, 1.36)	− 0.82 (− 8.19–6.17)	0.01*
Median (IQR) height (z score)***	− 1.7 (− 4.6, 1.9)	− 0.74 (− 8.14–6)	0.01*
Body mass index (z score)***	− 0.4 (− 3.5, 18.9)	− 0.54 (− 3.65–7.5)	0.55
*Staphylococcus aureus colonization* (MSSA + MRSA)** *n* (%)	25 (29.2)	529 (27.2)	0.86
Pseudomonas aeruginosa colonization** *n* (%)	32 (36.8)	311 (16.1)	0.01*
rhDNaz** *n* (%)	58 (66.7)	1694 (87.7)	0.01*
Pancreatic enzyme** *n* (%)	76 (87.5)	1663 (85.4)	0.56
Multivitamin** *n* (%)	77 (88.5)	1558 (80.7)	0.13
F508del** *n* (%)	20 (22.2)	486 (24)	0.91
Exon 2–3 deletion *n* (%)**	7 (9.7)	29 (1.5)	0.01*

*NCFRT* National CF Registry in Turkey, *rhDNase* recombinant human deoxyribonuclease, *NBS* newborn screening, *MSSA* methycilline sensitive *Staphylococcus aureus*, *MRSA* methycilline resistance *Staphylococcus aureus*

***One sample *t*-test; **Chi-square test; *Significant difference at 0.05 level

## Discussion

The purpose of this study was to assess the demographic information and CFTR mutations of refugee children with CF in Turkey and compare the findings with those of patients in the NCFRT. Refugee children were also successfully included in the NBS program in Turkey, a program that has not yet been introduced in most Arab countries like Syria. Even though refugee newborns had the same access to NBS as Turkish newborns, the diagnosis of refugee patients occurred at a later stage compared with Turkish patients. Though both groups had similar access to CF medications, we noticed a higher instance of pseudomonas colonization and poorer nutritional status in refugee patients during long-term follow-up. Because a significant amount of genetic diversity has been found in studies conducted among Arabs, it is advisable to conduct thorough CFTR analysis, including whole sequence analysis, and if needed, MLPA analysis, in patients suspected to have CF. Identifying mutations is vital for diagnosing diseases, obtaining appropriate modulator treatment options for relevant mutations, and providing genetic counseling to the affected family. It also aids in better understanding the relationship between genotype and phenotype.

Awareness of CF is low in Middle Eastern and Asian countries, and there is currently no known incidence [11]. The high rate of consanguineous marriage particularly among Arabs may contribute to a higher prevalence of CF. Among Arabs, the rate of consanguineous marriage is 50% in the general population but rises to 85% in families with CF [12]. The percentage of consanguineous marriages in Turkey is 24% [13]. As a result, the frequency of rare mutations also increases among Arabs [12]. In our study, the rate of consanguineous marriage was very high at 59%, and based on our data, the estimated incidence of CF among refugee Arab patients is 1/33,000. However, because our study did not include adult patients with CF and unregistered refugees, this information may not fully represent the CF population among refugee Arabs.

Compared with Western countries, CF survival is lower in Middle Eastern countries due to the availability of NBS programs, the introduction of promising drugs such as modulatory therapies, and improvements in care conditions [14]. Due to the lack of a NBS program and the low awareness among physicians, the average diagnosis is around age 3 years [15]. Although the median age of diagnosis for patients was significantly higher compared to NCFRT, patients born after 2015, who are eligible for the NBS program, were diagnosed more quickly. We hypothesized that this difference might be attributed to the challenges of living in and adjusting to a new country. However, implementing a well-structured NBS program in Arab countries could result in early identification of these patients, leading to improved prognosis and increased survival rates.

Multivitamin and pancreatic enzyme use rates of refugee patients were similar to NCFRT; only recombinant human deoxyribonuclease (hDNaz) use was found to be significantly lower in the refugee patient group. This outcome might be related to the significantly higher median age at the current age in the NCFRT group compared with the refugee patient group. Modulator treatments are not yet reimbursed for any patients with CF in Turkey. The Ministry of Health covers all healthcare expenses, except for modulator treatment, so accessing medicine is not a problem for refugee patients [16]. However, we observed that refugee patients with CF had lower height and weight z scores, as well as a higher rate of *P. aeruginosa* colonization, during their follow-up. Compared with NCFRT, the nutritional status and *P. aeruginosa* colonization of refugee patients with CF were found to be significantly worse. We believe that these results may be attributed to the challenging social living conditions faced by refugee patients, such as crowded family living, housing problems, poor dietary intake, and cultural differences.

In many studies conducted in Arab ethnic groups such as Jordan, Lebanon, Palestine, and Tunisia, F508del was found to be the most common mutation, with rates varying between 34 and 56% [17–20]. Two studies were conducted in Syria to evaluate CFTR mutations among CF patients. The first study included 25 patients, while the second study included 175 patients. In both studies, the most common mutation observed was F508del, accounting for 18% and 36% of the cases, respectively [21, 22]. Similar to reference studies, in our study, F508del was the most frequently detected mutation in the refugee patient group with a rate of 22%. This rate was 80.3% in the 2021 European Cystic Fibrosis Registry [23]. Global investigation showed that the frequency of the F508del mutation decreases from northern Europe, with Denmark having a rate of 87.2%, to southern Europe, with Italy having a rate of approximately 20% [23]. Based on geographic factors, it is expected that the frequency of F508del in Syria would be lower or equal to its frequency in Turkey [24]. Similar to this study, the most frequently detected F508del mutation in the 2021 NCFRT was reported as 24% [5].

In our study, we found that there was a high level of variants in CF mutations within the patient population. The second most common mutation was the deletion of exons 2–3. Interestingly, the frequency of this specific mutation in our study was significantly higher than the NCFRT and worldwide average [5, 25]. These findings suggest that the presence of exon 2–3 deletions in this population may be indicative of a common, founder mutation among Syrian patients.

In another study conducted on Iranian patients with CF, the most prevalent mutation was R334W, which was found at a rate of 40.74%, followed by F508del at a rate of 22% [26]. Another study conducted on patients with CF in the United Arab Emirates (UAE) revealed that the most common mutation was S549R, accounting for 28% of cases [27]. In a comprehensive study that examined 72 studies conducted in 22 countries, a total of 5481 Arab patients with CF were analyzed for CFTR genetic mutations. The study reported that the most frequently observed mutation was F508del, except for Iraq, Sudan, and Qatar. Furthermore, in Saudi Arabia, the most common mutations are c.3700A>G, 3120+1G>A, c.3909C>G, and c.1657C>T, whereas in Bahrain, the common mutations are c.2043delG, c.548A>T, c.4041C>G, and F508del. The c.3700A>G mutation is particularly prevalent among Gulf Cooperation Council countries [28–30]. Each ethnic group may have its own unique frequency of CFTR mutations. The results indicate that it is important to consider a different approach to molecular genetics diagnostic strategies in Arabic countries. Therefore, knowing the frequency and distribution of CF mutations in each population can be beneficial for managing the disease, developing diagnostic tools, and conducting prenatal diagnosis [31–33].

The strength of this study is that it implemented the NBS program for the first time in Syrian Arab refugee patients, leading to early diagnosis. However, despite free access to CF medications, no improvement in their malnutrition was observed during long-term follow-up. This study has some limitations. The high genetic heterogeneity made it impossible to establish the genotype-phenotype relationship of the patients. Language differences and communication difficulties were major obstacles in the follow-up and treatment of refugee patients [3, 4, 34]. Patients who were seeking asylum and immigrants could not be included in the study, so we may not have been able to reach all patients. Another limitation was the lack of data due to the retrospective nature of the study.

In conclusion, we found that the patients were diagnosed shortly after NBS which was successfully used on Arab refugees. Despite there being no issues with accessing CF medications, we noticed that their nutritional status was worse and that pseudomonas colonization increased during follow-up. We discovered significant genetic diversity and uncommon mutations in these refugees. Therefore, it is important to implement NBS for CF in Arab countries. Expanding global newborn screening programs and enhancing the living conditions for patients who had to migrate to other countries will have a positive impact on the progression of the disease.

## Data Availability

Available upon request to the corresponding author.
